# Accessibility to Specialized Public Oral Health Services from the Perspective of Brazilian Users

**DOI:** 10.3390/ijerph13101026

**Published:** 2016-10-19

**Authors:** Ricardo Dias de Castro, Marianne de Lucena Rangel, Marcos André Azevedo da Silva, Brunna Thaís Lucwu de Lucena, Alessandro Leite Cavalcanti, Paulo Rogério Ferreti Bonan, Julyana de Araújo Oliveira

**Affiliations:** 1 Programa de Pós-Graduação em Odontologia, Universidade Federal da Paraíba, João Pessoa 58051-900, PB, Brazil; mariannerangel01@gmail.com (M.d.L.R.); pbonan@yahoo.com (P.R.F.B.); julyana86@hotmail.com (J.d.A.O.); 2 Centro de Ciências da Saúde, Departamento de Fonoaudiologia, Universidade Federal da Paraíba, João Pessoa 58051-900, PB, Brazil; brunnaluckwu@hotmail.com; 3 Centro de Ciências da Saúde, Graduação em Odontologia, Universidade Federal da Paraíba, João Pessoa 58051-900, PB, Brazil; marcosandre.1991@hotmail.com; 4 Programa de Pós-Graduação em Odontologia, Universidade Estadual da Paraíba, Campina Grande 58429-500, PB, Brazil; dralessandro@ibest.com.br

**Keywords:** health services evaluation, health policy, dental health services

## Abstract

The Specialized Dental Clinics (SDCs) represent the first government initiative in Latin America aimed at providing specialized oral health services. This study sought to evaluate the organizational accessibility to specialized oral health care services in Brazil and to understand the factors that may be associated with accessibility from the user’s perspective. This epidemiological, cross-sectional and quantitative study was conducted by means of interviews with individuals who sought specialized public oral health services in the city of João Pessoa, Paraíba, Brazil, and consisted of a sample of 590 individuals. Users expressed a favorable view of the classification and resolutive nature of specialized services offered by Brazilian public health. The binary logistic regression analysis revealed weak points highlighting the difficulty involved in obtaining such treatments leading to unfavorable evaluations. In the resolutive nature item, difficulty in accessing the location, queues and lack of materials and equipment were highlighted as statistically significant unfavorable aspects. While many of the users considered the service to be resolutive, weaknesses were mentioned that need to be detected to promote improvements and to prevent other health models adopted worldwide from reproducing the same flaws.

## 1. Introduction

The study of the current organization and delivery of health services in Latin America involves an understanding of political and social contexts, which are strongly influenced by historical factors, marked by the state’s absence in the formulation of public health policies geared toward the interests of the majority. The re-democratization process of the continent’s main countries seeks to overcome social inequalities, such as problems involving agrarian issues, indigenous people, high population densities in major cities and the precariousness of respect for human rights. These aspects are closely related to the concept of health and are translated into health planning and practices. Understanding health services in this context represents an interdisciplinary field of study of great international importance [1].

In Brazil, the 1980s marked the resumption of the democratic process, which was accompanied, at the beginning of the 1990s, by the institutionalization of public health services in the country, characterized mainly by a universal, comprehensive and equitable nature. Oral health services go hand in hand with this process and therefore face the challenge of implementing actions that can respond to the demands of society, characterized by precariousness, high prevalence of oral disease and lack of access to health services [2,3]. 

In 2004, the Brazilian government adopted the National Oral Health Policy (Política Nacional de Saúde Bucal), which aims to reorganize primary health care services, with targeted treatment for families based on epidemiological indicators [4]. In addition, Specialized Dental Clinics (SDCs) were created. These clinics are intended for individuals referred by primary care services and mainly offer treatments in surgery, diagnosis, molding and adaptation of prostheses, periodontics, endodontics and for patients with special needs [5]. The SDCs represent the first government initiative in Latin America aimed at providing specialized oral health services. Therefore, understanding these services, whether from an organizational or political perspective, is extremely important because of their public and populational nature—as the majority of the population uses them—and because they are innovative, in that they represent the first government initiative in the sector.

This time of change means that the evaluation of health services, especially those of a public nature, should be a paramount, recommended and routine practice [6], both in developed [7,8,9] or developing [10] countries. These evaluations contribute to the development of strategies aimed at the qualification of treatment, rationalization of public funds and are considered a health surveillance practice [11], which should incorporate elements relating to the political, social and cultural organization of each location. 

With regard to access to oral health services, it is known that socioeconomic status [12,13], living conditions [14] and experience of caries [15,16] negatively affect the search for dental care. However, factors inherent to the organization of health services are poorly studied, especially those regarding specialized dental care. This fact gave rise to the present study, which aims to evaluate the organizational accessibility to specialized oral health care services in Brazil from the user’s perspective. 

## 2. Materials and Methods

### 2.1. Type of Study

This epidemiological, cross-sectional, quantitative study sought to better understand factors relating to the access to specialized public oral health services in the city of João Pessoa, Paraíba, Brazil, which is located in the northeast of the country and has unfavorable social indicators, with a Human Development Index (HDI) of 0.763.

### 2.2. Sample

Between August 2012 and December 2013, adults seeking any of four specialized public service dental practices were interviewed during the morning, afternoon or night shift, thereby diversifying the user profile and ensuring populational representativeness. The sample, defined according to statistical criteria, considered the prevalence estimate of the investigated event, coupled with a relative margin of error of 3%, a confidence level of 95% and a 20% non-response rate. Thus, considering that the variable analyzed in this study—difficulty in obtaining dental care in specialized services—has an estimated prevalence of 13% in Northeast Brazilian adults [17], a sample of 590 individuals was deemed to have enough power for the proposed research.

### 2.3. Data Collection

Data were collected using a structured questionnaire that addressed aspects relating to socioeconomic status, access to services, difficulties with regard to consultations and examinations and type of requested specialization. 

### 2.4. Calibration and Pilot Study

The researchers took part in technical training to establish standards for analysis and application of the questionnaire. To that end, a pilot study was conducted with 30 subjects who were not included in the final sample.

### 2.5. Variables

The dependent variables in the study were grouped into two categories: classification of oral health services offered by the SDC (both favorable and unfavorable) and the resolutive nature of oral health services offered by the SDC (both favorable and unfavorable). Table 1 shows the described distribution.

### 2.6. Statistical Analysis

The data collected were entered and processed using Statistical Package for the Social Sciences 22.0 software (Chicago, IL, USA) and were subjected to descriptive and inferential statistical analyses. The independent variables were dichotomized for bivariate analysis (Fisher’s exact test), followed by binary logistic regression to determine the factors with the greatest effect on access to specialized oral health services, considering α = 5%.

### 2.7. Ethical Aspects

The study was approved by the Research Ethics Committee of the Federal University of Paraíba with project identification code 69735 (data of approval: 2 September 2015), ensuring the participant’s anonymity and privacy.

## 3. Results

### 3.1. Descriptive Statistics

Characterization of the sample revealed that most subjects were female, less than or equal to 44 years of age and had an educational level ranging from completed high school education to higher education. Most respondents were characterized as economically active and as belonging to the low or middle-low class. 

### 3.2. Classification of Oral Health Services Offered by the SDC

Table 2 shows a bivariate analysis of user perceptions as to the classification of health services provided by the SDC in terms of whether they were favorable or unfavorable. There were no significant differences according to interviewees’ gender, age, education, employment and even socioeconomic status. Whether the SDC was located in the center or outskirts of the city did not significantly influence categorization of the service, which was considered favorable by most users, according to the numerical distribution of the sample.

Binary logistic regression analysis when the user’s perception was unfavorable in relation to the health service (Table 3) revealed that “difficulty getting specialized dental care” was the most cited complaint among interviewees when negatively characterizing the SDC’s service (odds ratio [OR] = 4.69; *p* < 0.001). Other aspects were also referenced by users, such as care disqualifiers (inadequate location, difficult scheduling, inconvenient opening hours, lack of medicines and inadequate infrastructure), but these factors were not statistically significant based on the sample composition.

### 3.3. Resolutive Nature of Oral Health Services Offered by the SDC

Regarding the resolutive nature of services provided in the specialized dental clinics, the majority were satisfied as to problems relating to oral health being resolved (positive classification), with no significant differences when considering dimensions such as gender, age and sociodemographic aspects (Table 4).

Although most respondents considered the resolutive nature of oral health problems in the SDCs as favorable, a binary logistic regression analysis of unfavorable perceptions of the service’s resolutive nature was performed (Table 5). Regarding sociodemographic aspects, it was observed that economically active respondents characterized the service’s resolutive nature as insufficient twice as often as non-active individuals (OR = 2.21, *p* = 0.032). Difficulty in accessing locations where SDCs were established also negatively affected users’ views (*p* = 0.049), as did the existence of queues (*p* = 0.008) and the absence of materials and equipment (*p* = 0.007).

## 4. Discussion

Recent studies have clearly emphasized the current gender difference with regard to seeking health care and accessibility to the same, with work being the main aspect explaining men’s greater absence or difficulty [18]. It is worth noting that the concept of “work” is related to both the fear of revealing weaknesses in a person’s work context and the argument that the services themselves do not have longer opening hours [19]. In the present study, males formed the smaller part of the sample, corroborating findings in the literature regarding differences in accessibility to services between men and women. It also appears that women had a higher proportion of public service usage, when sought, while men used private services more often [20].

Judgment of the quality of service provided in the SDC revealed no significant differences when evaluated by respondents of different genders, education levels, ages and social classes. These dimensions did not have a significant effect on service classification, which was said to be favorable by most respondents. Scientific research evaluating specialized oral health services in Brazil is scarce. In a study on specialized dental clinics in the city of Recife, in the state of Pernambuco (PE), situated in the northeast of Brazil, service quality was analyzed, and respondents’ evaluations of the quality dimensions were largely positive, especially when related to human relations between the dentist and assistant, efficacy/discomfort after treatment, technical and scientific quality or technology of equipment and acceptability/explanation of treatment by the professional [21].

When these positive results are compared to studies evaluating SDC services from other perspectives, differences become apparent. In an evaluation performed by [11] on the productivity performance of 613 SDCs, by measuring the overall achievement of targets with data collected in the Outpatient Health Information System (Sistema de Informações Ambulatoriais em Saúde—SIA/SUS), SDC performance was bad in most regions and was classified as bad and very bad in 45.6% of cases in the Northeast of Brazil, where the SDCs involved in the present study are located. 

Such disagreement can be explained by the differences in the populations used to obtain the data. Studies focusing on people’s opinions have a subjective nature, as subjectivity is inherent to the different perceptions of each individual when evaluating whether something met his/her expectations, along with psychosocial factors and the effects of previous experiences as references. Using factor analysis [22], verified that 90%–95% of patient satisfaction variations were related to variables inherent to the patients themselves, such as age, morbidity and previous experiences. Another study highlights other influencing factors, such as satisfaction with life in general and with the community in which he/she resides, the level of the professional’s credibility, increased feelings of internal control (the individual is considered responsible for his own life), rather than non-external control (the individual imagines her life to be controlled by factors external to her person), and the user’s current state of health [23].

In developed countries such as the United States, the quality rate of urban oral health services evaluated by users exceeds 90%, although difficulties relating to access can be identified, especially among the uninsured, particularly for dentistry, oral surgery, and dental prosthesis services [8]. However, rural American populations use fewer oral health services, though they have higher amounts of dental problems and less access to fluoridated water. These factors influence the geographic distribution of the services [24].

The mere existence or availability of a health service does not guarantee accessibility. It is necessary to subdivide this concept into geographical accessibility (distance, time of transportation and means of transport should determine the location of establishments, rather than theoretical jurisdiction areas), affordability (possible payments or contributions for using the service should not constitute obstacles), cultural accessibility (there should be no conflict between the technical and administrative standards of services and the habits, cultural patterns and customs of the communities in which they are provided) and functional accessibility (the services should be provided expediently and on an ongoing basis and should be available at any time, meet real demand and include a reference and counter-reference system to ensure easy access to the level of assistance required) [25].

From the point of view of this broad concept of accessibility, it can be observed that users report difficulty in accessing specialized care in these centers. This factor is the most significant and critical aspect in the formation of the respondents’ negative opinions (OR = 4.69; *p* < 0.001). With regard to the resolutive nature aspect of oral health problems, the existence of queues was statistically significant as a negative aspect (*p* = 0.008). These data are similar to those observed in other studies conducted in Brazil [21,26]. Such difficulties in obtaining appointments are a reality of public Brazilian health services, which are characterized by long queues, indicative of the fragility of the universal character advocated by the country’s own public health policy, leading those with better socioeconomic conditions to seek private services. This fact is evidenced by the economic classification indicator, which found that more than 80% (*n* = 478) of users of these services belong to the lower or lower-middle class. In this respect, it is interesting to note the possible relationship with the good results presented, as despite the fact that an association between satisfaction and income has not been identified in the international literature, in Brazil, there seems to be an inverse correlation, with users from more popular social classes being more likely to report being satisfied with services [27]. 

Research involving the SDCs in Brazil has shown that the smaller the city and the lower the HDI, the worse the care performance observed in specialized dental clinics. The same study also showed that there were striking inequalities in the perception of dental care needs and access to SDCs, the latter being greater for women, middle-aged adults and individuals with better education [26]. Although the municipality evaluated in the present study has an unfavorable HDI, the quality of service access was reported as positive by most users. This finding may be attributed to better distribution of specialized professionals in the country’s capitals compared to rural areas [25].

Regarding the perception of the resolutive nature of care provided in the SDCs, the majority of respondents had a positive view and considered the service provided favorably. Similar data were observed in Recife, Brazil, where based on the evaluation of the resolutive nature dimensions in SDCs, it was found that users of these services were satisfied regarding appearance and chewing ability, confirming the findings of the present study [21]. No association was established between education or social level and classifying outcomes as favorable. In this context, we must also consider that improving access to private or insured dental treatment can be a determining factor in masking the perception of problems in accessing public health services, which should provide sufficient resources to ensure the universality of treatment. The current belief still lies on the assumption that a service considered “free” by the majority of the population can generate bias in the perception of quality, when disregarding taxes and other sources of income that should be targeted at the qualification of the service.

However, as can be observed in Table 5, among individuals who considered the service’s resolutive nature as unfavorable, those who reported being professionally active tended to judge the SDCs’ caries resolutive nature capacity negatively. Other dimensions were cited as determinants of the lack of resolutive nature, such as incorrect location, length of queues, lack of materials and broken equipment. Such aspects corroborate the findings found by De Goes et al. [11], where SDC professionals encountered difficulties in meeting the recommended care goals set out by the government, as it was shown that more requirements needed to be implemented in the North and Northeastern SDCs.

Overall, despite the failures reported by users regarding access and the resolutive nature of SDC dental services, respondents expressed satisfaction with the service. This finding can be attributed to attempts to improve service management in light of the rapid and thorough Unified Health System (Sistema Único de Saúde—SUS) data capture, which in turn gives managers indispensable decision-making material [11]. Another important aspect is the promotion of the guarantee of a comprehensive approach to access to primary care and continuity of care in specialized treatment, which is made possible with the involvement of the entire team in the implementation of protocols and induction projects, reinforcing the importance of building reference and counter-reference processes, which are still insufficient in the health care networks [17].

However, it is essential that studies aimed at better understanding the work process in specialized dentistry care services are conducted to make it possible to understand the organizational structure, working relationships, planning, scheduling and impacts of actions on user health, considering locoregional differences, whether related to the municipality’s size, coverage area (urban and rural areas), and social and cultural characteristics.

## 5. Conclusions

The vast majority of users had a favorable opinion of the service quality in the SDCs, and there were no associations between respondents’ perceptions and gender, age or socioeconomic status. While many users considered the service resolutive, weaknesses were mentioned relating to access, existence of queues and lack of materials, supplies or infrastructure.

## Figures and Tables

**Table 1:** Dependent and independent variables investigated.

Dependent Variables	Independent Variables
Social classification Income Education Occupation	Service evaluation Type of service sought Waiting time for 1st consultation Waiting time for last consultation Referral for examination Difficulties in performing examinations Intending to return to primary care Means of transport to get the SDC Geographical access

SDC: Specialized Dental Clinic.

**Table 2:** Bivariate analysis (Fisher’s exact test) of users’ perceptions in relation to oral health services of the Specialized Dental Clinics (SDC) and sociodemographic variables (*n* = 589).

Variable/Dimensions		Classification of SDC’s Oral Health Services		*p*-Value
		Favorable	Unfavorable	
SDC where the interview was held	Center	229	36	0.76
	Outskirts	262	62	
Gender of interviewee	Male	173	38	0.56
	Female	318	60	
Age of interviewee	Up to 44 years	314	70	0.165
	Over 44 years	177	28	
Education level	Up to complete elementary	189	32	0.305
	From high school to higher	302	66	
Profession/work of interviewee	Economically active	266	52	0.736
	Not economically active	207	44	
Social classification	Favorable	93	18	1.000
	Unfavorable	398	80	

**Table 3:** Binary logistic regression analysis of users’ unfavorable perceptions regarding the Specialized Dental Clinics (SDC)’s oral health services and sociodemographic and service-related variables (*n* = 589).

Variable/Dimensions		Statistical Evaluation		*p*-Value
		OR	CI (95%)	
Age of interviewee	Up to 44 years	1.4378	0.742–2.782	0.283
	Over 44 years	1		
Location difficult to access	Yes	1.148	0.375–3.514	0.809
	No	1		
Making an appointment is difficult	Yes	1.306	0.461–3.703	0.615
	No	1		
There are queues	Yes	1.781	0.812–3.908	0.150
	No	1		
Opening hours do not meet my needs	Yes	0.606	0.208–1.768	0.359
	No	1		
Lack of medication	Yes	1.538	0.460–5.144	0.485
	No	1		
Difficulty in getting dental treatment	Yes	4.659	2.212–9.814	**<0.001**
	No	1		
Missing material or broken equipment	Yes	0.846	0.373–1.922	0.690
	No	1		
Lack of supervision	Yes	0.739	0.000	1.000
	No	1		
The service or treatment I need is not available	Yes	3.903	0.567–26.852	0.166
	No	1		
Not well accepted	Yes	0.008	0.000	1.000
	No	1		
Were you made welcome in the SDC?	Yes	0.308	0.056–1.698	0.176
	No	1		
Long treatment waiting time	Yes	1.719	0.770–3.836	0.186
	No	1		
Service only with form	Yes	<0.001	0.000	0.998
	No	1		

The statistically significant *p*-Values are represented in bold.

**Table 4:** Bivariate analysis (Fisher’s exact test) of users’ perceptions in relation to the resolutive nature of the Specialized Dental Clinics (SDC)’s oral health services and sociodemographic variables (*n* = 589).

Variable/Dimensions		Resolutive Nature of SDC Oral Health Services		*p*-Value
		Favorable	Unfavorable	
SDC where interview took place	Center	244	21	0.559
	Suburb	293	31	
Gender of interviewee	Male	190	21	0.545
	Female	347	31	
Age of interviewee	Up to 44 years	348	36	0.648
	Over 44 years	189	16	
Education level	Up to complete elementary	206	15	0.230
	From high school to higher	331	37	
Profession/work of interviewee	Economically active	281	37	**0.007**
	Not economically active	238	13	
Social classification	Favorable	97	14	0.136
	Unfavorable	440	38	

The statistically significant *p*-Values are represented in bold.

**Table 5:** Binary logistic regression analysis of users’ unfavorable perceptions in relation to the resolutive nature of oral health services and sociodemographic and service-related variables (*n* = 589).

Variable/Dimensions		Statistical Variables		*p*-Value
		OR	CI (95%)	
Profession/work of interviewee	Economically active	2.21	1.070–4.600	**0.032**
	Not economically active	1		
Social classification	Favorable	1.143	0.512–2.551	0.744
	Unfavorable	1		
Location is difficult to access	Yes	3.454	1.003–11.894	**0.049**
	No	1		
Making appointments is difficult	Yes	1.517	0.413–5.572	0.531
	No	1		
Difficult to get dental treatment	Yes	1.197	0.471–3.042	0.706
	No	1		
There are queues	Yes	3.685	1.411–9.621	**0.008**
	No	1		
Opening hours don’t meet my needs	Yes	0.778	0.238–2.545	0.678
	No	1		
Lacking medication	Yes	0.741	0.129–4.261	0.737
	No	1		
Lacking material or broken equipment	Yes	3.817	1.449–10.056	**0.007**
	No	1		
The service or treatment I need is not available	Yes	0.603	0.048–7.497	0.694
	No	1		
Not well accepted	Yes	<0.001	0.000	0.999
	No	1		
Were you made welcome in the SDC?	Yes	0.633	0.84–4.779	0.658
	No	1		
Long treatment waiting time	Yes	0.596	0.201–1.765	0.350
	No	1		

The statistically significant *p*-Values are represented in bold.

## References

[1] Labra M.E. (2001). Política e saúde no Chile e no Brasil: Contribuições para uma comparação. Ciência Saúde Coletiva.

[2] Antunes J.L.F., Narvai P.C. (2010). Políticas de saúde bucal no Brasil e seu impacto sobre as desigualdades em saúde. Revista de Saúde Pública.

[3] Roncalli A.G. (2006). Epidemiologia e saúde bucal coletiva: Um caminhar compartilhado. Ciência Saúde Coletiva.

[4] Nascimento A.C., Moyses S.T., Werneck R.I., Moyses S.J. (2013). Oral health in the context of primary care in Brazil. Int. Dent. J..

[5] Soares C.L. (2012). Constructing public oral health policies in Brazil: Issues for reflection. Braz. Oral Res..

[6] Cameron A., Lart R., Bostock L., Coomber C. (2014). Factors that promote and hinder joint and integrated working between health and social care services: A review of research literature. Health Soc. Care Community.

[7] Widstrom E., Komu M., Mikkola H. (2013). Longitudinal register study of attendance frequencies in public and private dental services in Finland. Community Dent. Health.

[8] Jones E., Shi L., Hayashi A.S., Sharma R., Daly C., Ngo-Metzger Q. (2013). Access to oral health care: The role of federally qualified health centers in addressing disparities and expanding access. Am. J. Public Health.

[9] McKernan S.C., Kuthy R.A., Momany E.T., McQuistan M.R., Hanley P.F., Jones M.P., Damiano P.C. (2013). Geographic accessibility and utilization of orthodontic services among medicaid children and adolescents. J. Public Health Dent..

[10] Kahabuka C., Moland K.M., Kvåle G., Hinderaker S.G. (2012). Unfulfilled expectations to services offered at primary health care facilities: Experiences of caretakers of underfive children in rural Tanzania. BMC Health Serv. Res..

[11] De Goes P.S.A., Figueiredo N., das Neves J.C., da Motta Silveira F.M., Costa J.F.R., Pucca Júnior G.A., Rosales M.S. (2012). Avaliação da atenção secundária em saúde bucal: Uma investigação nos centros de especialidades do Brasil. Cadernos de Saúde Pública.

[12] Crocombe L.A., Broadbent J.M., Thomson W.M., Brennan D.S., Slade G.D., Poulton R. (2011). Dental visiting trajectory patterns and their antecedents. J. Public Health Dent..

[13] Kengne Talla P., Gagnon M.P., Dramaix M., Leveque A. (2013). Barriers to dental visits in Belgium: A secondary analysis of the 2004 national health interview survey. J. Public Health Dent..

[14] Wagner J., Diehl K., Mutsch L., Löffler W., Burkert N., Freidl W. (2014). Health status and utilisation of the healthcare system by homeless and non-homeless people in Vienna. Health Soc. Care Community.

[15] Kuthy R.A., Kavand G., Momany E.T., Jones M.P., Askelson N.M., Chi D.L., Wehby G.L., Damiano P.C. (2013). Periodicity of dental recall visits for young children first seen in community health centers. J. Public Health Dent..

[16] Noro L.R., Roncalli A.G., Mendes Júnior F.I., de Lima K.C., Teixeira A.K. (2014). Toothache and social and economic conditions among adolescents in northeastern Brazil. Ciência Saúde Coletiva.

[17] Souza G.C., Lopes M.L.D.S., Roncalli A.G., Medeiros-Júnior A., do Céu Clara-Costa I. (2015). Referência e contra referência em saúde bucal: Regulação do acesso aos centros de especialidades odontológicas. Revista de Saúde Pública.

[18] Gomes R., Schraiber L.B., Couto M.T., Valença O.A.A., Silva G.S.N., Figueiredo W.S., Barbosa R.M., Pinheiro T.F. (2011). O atendimento à saúde de homens: Estudo qualitativo em quatro estados brasileiros. Physis: Revista de Saúde Coletiva.

[19] Knauth D.R., Couto M.T., Figueiredo W.S. (2012). A visão dos profissionais sobre a presença e as demandas dos homens nos serviços de saúde: Perspectivas para a análise da implantação da política nacional de atenção integral à saúde do homem. Ciência Saúde Coletiva.

[20] Soares F.F., Chaves S.C.L., Cangussu M.C.T. (2015). Governo local e serviços odontológicos: Análise da desigualdade na utilização. Cadernos de Saúde Pública.

[21] Lima A.C.S., Cabral E.D., Vasconcelos M.M.V.B. (2010). Satisfação dos usuários assistidos nos centros de especialidades odontológicas do município do Recife, Pernambuco, Brasil. Cadernos de Saúde Pública.

[22] Sixma H.J., Spreeuwenberg P.M., van der Pasch M.A. (1998). Patient satisfaction with the general practitioner: A two-level analysis. Med. Care.

[23] Weiss G.L. (1988). Patient satisfaction with primary medical care. Evaluation of sociodemographic and predispositional factors. Med. Care.

[24] Skillman S.M., Doescher M.P., Mouradian W.E., Brunson D.K. (2010). The challenge to delivering oral health services in rural America. J. Public Health Dent..

[25] Bulgareli J.V., Faria E.T., Ambrosano G.M.B., Vazquez F.L., Cortellazzi K.L., Meneghim M.C., Mialhe F.L., Pereira A.C. (2013). Informações da atenção secundária em odontologia para avaliação dos modelos de atenção à saúde. Revista de Odontologia da UNESP.

[26] Peres M.A., Iser B.P.M., Boing A.F., Yokota R.T.C., Malta D.C., Peres K.G. (2012). Desigualdades no acesso e na utilização de serviços odontológicos no brasil: Análise do sistema de vigilância de fatores de risco e proteção para doenças crônicas por inquérito telefônico (vigitel 2009). Cadernos de Saúde Pública.

[27] Santos M.P. (1995). Avaliação da qualidade dos serviços públicos de atenção a saúde da criança sob a ótica do usuário. Revista Brasileira de Enfermagem.

